# Mortality after road traffic crashes in a system with limited trauma data capability

**DOI:** 10.1186/1752-2897-8-4

**Published:** 2014-02-13

**Authors:** Hassan Saidi, Ben Kasyoka Mutiso, Julius Ogengo

**Affiliations:** 1 Department of Human Anatomy, University of Nairobi, Riverside Drive, P.O. Box 30197, Nairobi 00100, Kenya; 2 Embu Provincial General Hospital, P.O. Box 33, Embu 60100, Kenya

## Abstract

**Background:**

Africa has 4% of the global vehicles but accounts for about one tenth of global vehicular deaths. Major trauma in Kenya is associated with excess mortality in comparison with series from trauma centers. The determinants of this mortality have not been completely explored.

**Objectives:**

To determine the factors affecting mortality among road users in Nairobi, Kenya.

**Methods:**

Cross-sectional study of prospectively collected data of trauma admissions at the Kenyatta National Hospital over a calendar year (2009–2010). Information collected included age, gender, road user type, principal anatomical region of injury, admission status, admission blood pressure and GCS, disposition destination, Injury Severity Score (ISS), injuries sustained, treatment and mortality at two weeks. Major or severe injury was defined as injuries of ISS > 15. Groups based on in-hospital survival were compared using determinants of mortality using *X*^2^ or students *t*-test as appropriate. Logistic regression was used to assess the independence of predictive variables.

**Results:**

One thousand six hundred forty seven (1647) patients were admitted for trauma during the study period. Traffic admissions were 1013 (61.7%) and males predominated (79.8%). The average age of patients admitted was 31.7 years. Pedestrians, vehicle occupants and motorcyclists represented 43.3%, 27.2% and 15.2% of the road users injured. The proportion of patients with ISS > 15 was 10.9%.

The overall mortality was 7.7%. Mortality for ISS > 15 was 27.6%. The following factors significantly predicted mortality on univariate analysis: head injury, abdominal injury, transfer in status, blood transfusion, ICU admission, age > 60 years, Glasgow coma scale (GCS) and injury severity. GCS (p = 0.001) and ISS > 15 (p < 0.05) remained significant predictors on regression analysis.

**Conclusion:**

Trauma mortality rates in this study exceed those from mature trauma systems. Head injury and injury severity based on the ISS are independent predictors of mortality after traffic trauma. Improvements in neurosurgical and critical care services ingrained within wider primary and secondary prevention initiatives are logical targets.

## Introduction

Road traffic injuries are a global public health problem, with an estimated 1.2 million deaths and 50 million non-fatal injuries per year [1]. Developing countries account for most of these deaths. Highly motorized countries have 60 per cent of global vehicles, but account for only 14 per cent of global deaths. Africa, with only 4% of the global vehicles, accounts for 11% of global deaths [2]. In Kenya, 68 deaths per 1,000 registered vehicles are recorded annually (3,000 deaths), a rate 30–40 times that in industrialized countries [3].

A 2000 study showed an unadjusted mortality rate of 35.6% for severe injuries resulting from road collisions in Kenya’s capital, Nairobi [4]. This rate is six times higher than that reported for countries with high income. The determinants of this mortality are incompletely documented. Since the 2000 study, access to advanced trauma life support (ATLS) protocols has improved and policy enhancement towards enforcement of traffic laws [5] embraced. We aimed to study the pattern of road trauma mortality from a hospital perspective and assess the determinants of this mortality using a larger sample of admissions over one calendar year.

## Methods

Kenyatta National Hospital (KNH) is the largest health facility in Kenya. It is the teaching hospital of the University of Nairobi and has a catchment population of about 3,000,000 people. The hospital receives most of the trauma in the city and its environs. The study sample included all admissions due to traffic between November 2009 and December 2010. The following factors were recorded: age, gender, road user type, admission from scene or transfer-in, anatomical region injured, Intensive Care Unit admission, systolic blood pressure on admission, Glasgow Coma Scale (GCS), Injury Severity Score (ISS), treatment and mortality at two weeks. Abbreviated Injury Scale categorization and calculation of Injury Severity Scores were done from the anatomic description of the injuries. Cost data referred to direct cost to the patient as captured from the hospital billing system. Costs are converted to USD from Kenya shillings (conversion rate: 86 shillings to one USD at the time). The mortality information was further evaluated for significant predictors and analyzed (with the Statistical Package for the Social Sciences (version 18, SPSS Inc. software) using the Chi square test, Fisher’s exact test and Student *t*-test as appropriate. A p-value of <0.05 was considered significant. Variables analyzed included head/neck involvement, age, gender, injury severity, need for major surgical procedure and transfusion. ISS > 15 was the threshold for severe injury. Major surgery entailed operations lasting more than one hour and included craniotomies, laparotomies, internal fixation of limb fractures, major wound debridements.

### Consent

Written informed consent was obtained from the patient for the publication of this report and any accompanying images.

### Ethical approval

Ethical approval was obtained from the KNH-UON ethical review committee and the work was done in compliance with the Helsinki Declaration.

## Results

**Figure 1:**
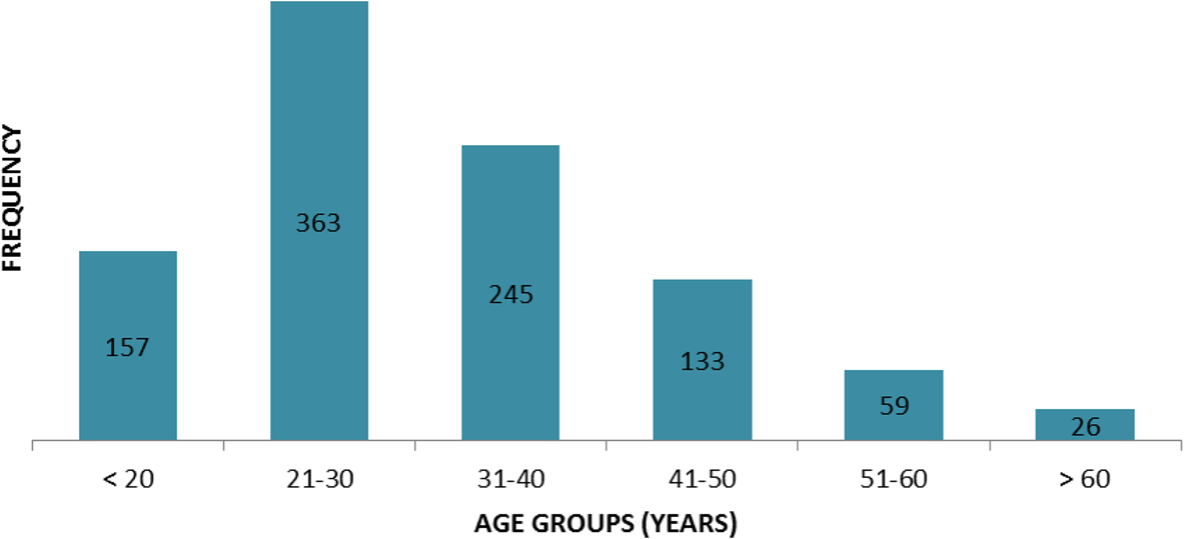
Age distribution for traffic injuries at KNH.

There were 1647 trauma admissions during the study period and 1013 (61.7%) were traffic related. Males predominated (82.4%). The average age of the patients was 31.7 years (range, 1-98 years, peak age 21–30 years) (Figure 1).

**Figure 2:**
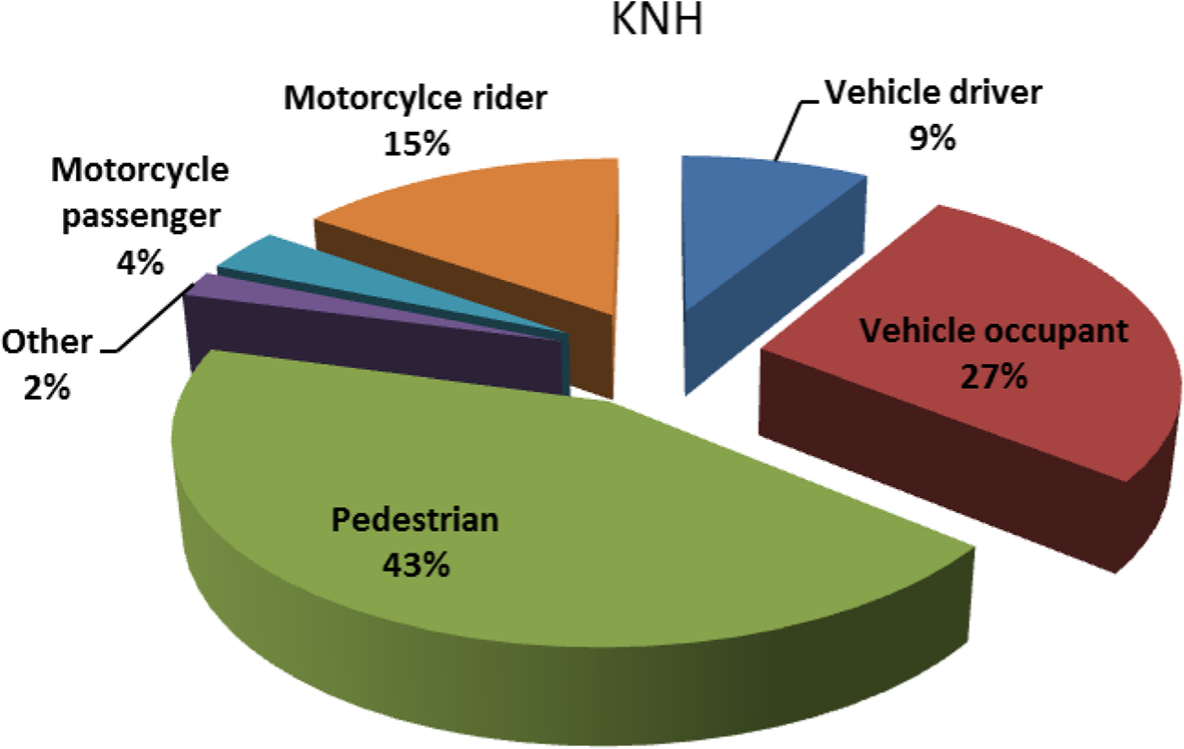
Road user categories in traffic trauma admissions.

The most common road user injured was the pedestrian (Figure 2) Seat belt use was recorded for 39.3% of motor vehicle drivers and 11.6% for occupants. Helmet use was reported for 18.2% of motorcycle passengers and 45.8% of riders.

Less than half of the patients (44.5%) were admitted directly from the crash scene while 55.4% were transferred from other facilities. The average Injury Severity Score was 7.27 ± 4.26 and the proportion with severe injury was 10.9% overall ((highest for motorcycle passenger) (Table 1)). Six point seven percent were disposed to the ICU from the accident and emergency department.

**Table 1:** Injury severity, length of hospital stay and direct costs by road user type

**Road user**	**Mean ISS**	**Proportion with ISS > 15**	**Mean LOS (days)**	**Mean cost (USD)**
Motor vehicle driver	7.2 ± 3.8	10.5%	29.7 ± 36.8	700.80 ± 57.51
Motor vehicle occupant	7.5 ± 4.6	15.2%	31.4 ± 43.6	507.87 ± 73.83
Pedestrian	7.0 ± 4.1	12.6%	33.0 ± 35.3	464.82 ± 50.31
Motorcycle passenger	7.3 ± 3.6	17.1%	33.4 ± 48.4	487.89 ± 61.84
Motorcycle rider	7.6 ± 4.0	15.8%	21.0 ± 26.2	301.84 ± 35.09

The most common anatomical regions injured were the extremity (56.4%) and head/neck (31.2%). The road users with the highest rate of extremity injuries were motorcycle occupants (62.9%). Extremity injury rate was 60.5% for MV driver, 46.23% for MV occupant, 60.1% for pedestrian and 61.8% for motorbike rider. The rate of head and neck injuries were 27.9%, 34.1%, 28.8%, 25.7% and 34.9% for motor vehicle drivers, motor vehicle occupants, pedestrians, motor cycle occupants and motor cycle riders respectively (Table 2). Of 315 injuries in the head/neck, 200 (63.5%) were isolated to the head, 28 (8.9%) isolated to the face and the rest were multiple injuries.

**Table 2:** Predominant region of injury versus road use category at KNH

**Injury**	**MVC driver (%)**	**MVC occupant (%)**	**Pedestrian (%)**	**Other (%)**	**MC occupant (%)**	**MC rider (%)**	**Total**
Head/neck	24 (7.7)	83 (29.7)	124 (38.6)	10 (3.25)	9 (16.9)	53 (16.9)	313
Chest	1 (3.1)	14 (43.8)	15 (46.9)	0	1 (3.1)	1 (3.1)	32
Abdomen	2 (6.1)	7 (21.2)	22 (66.7)	0	0	2 (6.1)	33
Spine	7 (14.6)	31 (64.6)	5 (10.4)	1 (2.1)	2 (4.2)	2 (4.2)	48
External	0	1 (12.5)	6 (75.0)	0	1(12.5)	0	8
Extremity	52 (9.2)	126 (22.4)	259 (46.0)	10 (1.8)	22 (3.9)	94 (16.7)	563
Total	86 (8.6)	272 (27.4)	431 (43.2)	21 (2.1)	35 (3.5)	152 (15.2)	997

The mean hospital stay was 30.4 ± 37.5 days (range 1–130, highest for motorcycle passenger).

Overall mortality was 7.7%. Mortality was 9.5% for motorcycle rider, 8.6% for MVC and motorcycle occupant, 7.5% for pedestrian and 3.6% for MVC driver.

Factors that were significantly associated with hospital mortality on univariate analysis were head involvement, ICU admission, non-surgical treatment and blood transfusion (p < 0.01). Additionally, mortality was significantly influenced by transfer status at admission (p = 0.01), abdominal injury (p = 0.011), injury severity score (p < 0.001) and GCS (p < 0.001). Age (p = 0.07), gender (p = 0.38) and admission BP (p = 0.96) and individual road user category did not influence mortality (Tables 3 and 4).

**Table 3:** Univariate analysis of factors influencing mortality after traffic trauma

**Variable**		**Alive**	**Died**	**P value**	**OR (C/I)**
Disposition	Wards	841	41 (4.6%)	< 0.001	10.1 (6.0-16.8)
	ICU/OR	71	35 (33.0%)		
Region of injury	Head/neck	263 (85.4%)	45 (14.6%)	< 0.001	3.2 (2.1 -4.9)
	Other	649 (95.4%)	31 (4.6%)		
Surgical treatment	Major procedure	452 (96.8%)	15 (3.2%)	< 0.001	3.9 (2.2-7.0)
	Nonsurgical care	446 (88.5%)	11.5%		
Injury severity	ISS ≤ 15	806 (95.4%)	39 (4.6%)	< 0.001	7.9 (4.8 – 12.9)-
	ISS > 15	97 (72.4%)	37 (27.6%)		
Admission status	Direct from scene	383 (95.1%)	20 (4.9%)	0.001	2.5 (1.5-4.3)
	Transfer-in	409 (88.5%)	53 (11.5%)		
Age	< 60 years	862 (92.9%)	66 (7.1%)	0.07	2.4 (0.9 – 6.5)
	≥ 60 years	27 (84.4%)	5 (15.6%)		
Gender	Male	718 (92.1%)	62 (7.9%)	0.38	1.3 (0.7-2.4)
	Female	185 (93.9%)	12 (6.1%)		
Blood product	Transfused	142 (82.6%)	30 (17.4%)	< 0.001	2.6 (1.7-4.1)
	Not transfused	641 (93.4%)	45 (6.6%)		
Specific injury	Head injuries	173 (84.6%)	31 (15.2%)	< 0.001	2.6 (1.7-4.0)
	No head injury	732 (94.2%)	45 (5.8%)		
	Abdominal injury	18 (78.3%)	5 (21.7%)	0.011	2.9 (1.3-6.6)
	No abdominal injury	887 (92.6%)	71 (7.4%)		

**Table 4:** Influence of mean ISS, BP, GCS and age on mortality for traffic injury

	**Alive**	**Died**	**P value (C/I of mean difference)**
**Age (years)**	31.68	33.85	0.21 (-5.5 – 1.2)
**Admission GCS**	14.01	10.35	< 0.001 (2.4 – 4.9)
**Admission BP**	117.01	117.11	0.96 (-4.5 -4.3)
**ISS**	6.88	11.58	< 0.001 (-5.6- -3.7)

## Discussion

This study has shown that road injuries continue to be the biggest component of the trauma burden at KNH and are associated with significant rate of death. In this study 7.7% of patients (43.6% of these pedestrians, 34.2% occupants, 22.0% of motorcycle users) died within fourteen days of admission. Although no age group was immune, the peak age of individuals in their third decade of life underscore the significant economic burden of road crashes [4]. The cost of the injury due to traffic injuries, computed from treatment costs and lost productivity, has been estimated to be equivalent to 1-2% of GDP of most countries [6].

The other results on trauma demography echo similar statistics in the trauma literature from low income countries where males, pedestrians and motorcyclists are most vulnerable [7]. The characteristics of the male thought to contribute significantly to the gender bias include a risk taking behavior and younger age. The predominant involvement of pedestrians and motorcyclists in this study differs from road user patterns in high income countries where vehicle occupants predominate. In the United Arab Emirates, a high income developing country, drivers are the most frequently injured road users. Middle to high income Emirati formed a big proportion of vehicle occupants while 88% of injured pedestrian were non-Emirati from low income countries [8]. Injury prevention initiatives locally should therefore prioritize pedestrian and motorcyclist safety through educational, engineering and legislative programs, proven to reduce road trauma events [7,9]. These may include enforcement of speed laws, motorcycle and pedestrian lanes, use of seat belts, helmets and general public education on traffic safety. Intervention targeting motorcyclists have shown that the use of helmets reduce trauma mortality [9] while protective attire including padded suits and boots may impact on the morbidity to the limbs that follow motorcycle crashes. Kenya recently experienced the evidence that the road carnage can indeed be controlled using local mechanisms modified from their proven track records in high income countries. When introduced for a short period in 2004, ‘Michuki laws’ that enforced traffic rules in the public transportation sector was associated with a reduction in hospital admission and numbers of fatal and serious injuries [5].

The average hospital stay was long (30.4 days) for our patients. Comparatively, hospital stay was significantly shorter (9.7 days) for patients in the UAE even with higher acuity trauma (a greater proportion needing ICU care (13% versus 6% in the current study) for UAE patients [8]. The explanation for the KNH statistics lies in the policy of fixation of skeletal fractures adopted. Most patients sustaining limb fractures cannot afford implants and the timing of fixation influenced by when families can raise enough funds to access implants. Rigorous cost-effectiveness assessment of this local resource dynamic must be undertaken so that an informed position on policy change to early fixation can be embraced. This policy reorientation must be guided by the potential economic productivity of the young patients who could go back to work earlier and that savings may be made by policy of early fracture fixation by reducing complications associated with prolonged hospitalization.

The overall and major trauma mortality was 7.7% and 27% respectively. This compares unfavorably with trauma mortality rate in high income countries. Although major trauma mortality has improved from a decade ago (36%), the current rate is still five times higher from rate in high income countries [10]. Differences in resources and advances in organization of trauma care may explain the disparate rates [11,12]. There is a relationship between a country’s level of income and mortality; higher incomes are associated with declining mortality [8,13]. This presumably must relate to levels of investments in the trauma care infrastructure. The specific investments have included maturation of trauma systems, enhanced protocols in trauma resuscitation including damage control surgery, lung protective ventilation and traumatic brain injury. Between 1996–2008 for example, mortality in one trauma center in the USA ranged from 3 to 3.7% even as the center handled a higher proportion of older patients and worse injuries than in our study [14]. Nairobi lacks an organized trauma system at the present moment. Serious efforts are needed to put in place a trauma care system that can optimize outcomes.

This study has shown that head involvement, GCS (GCS was 10.0 for non-survivors but 14.0 for survivors), nonsurgical treatment, blood transfusion, transfer-in status and injury severity were significant predictors of early mortality at univariate analysis but only low GCS and injury severity were independent predictors on logistic regression. The result on head injury and GCS is consistent with previous studies [4]. We contend that the other variables lost in the logistic regression are useful proxies to injury severity and head involvement. Admission to the ICU, blood transfusion, transfer-in and non-surgical status are associated with severe illness even in settings outside trauma. Patients with head injury are the more likely trauma victims to be transferred to KNH for specialized investigations and critical. There is need to study whether this is the case and strategies to improve head trauma services.

The result on blood pressure was surprising. Several other series have shown that this modifiable variable is a predictor of trauma mortality [8,15]. Possible explanations include the fact the study population was young (threshold for physiological derangements higher) and that this initial vital sign was not recorded for some patients. Although hypotension is reversible and could explain the result, we contend that sustained hypotension is a marker of injury severity and trauma protocols should still take the vital sign seriously.

The interactions of age, gender and road user type with mortality were not significant. This is also consistent with other studies. The implication of this is that all trauma should continue to be treated along similar lines initially (primary survey) for both gender and mechanisms.

This study had limitations. As stated earlier, the manner in which skeletal injury is managed at KNH limits comparisons with trauma centers. Secondly, this was a non-registry data analysis and is tedious. To enable the institution continuously interrogate its trauma performance, a registry system should be developed to enhance continuous evaluation of care. We have not analyzed the risk factors for road trauma including alcohol, speeding and restraint, known for their impact on road trauma mortality [16] and important ingredients for the development of prevention measures.

In conclusion, the trauma mortality rate at KNH is comparatively high. The independent predictors of mortality are GCS and injury severity score. The effects of neurotrauma and complex injury can be mitigated by developing a responsive trauma system including a registry system and critical care.

## Competing interests

The authors declare that they have no competing interests.

## Authors’ contributions

HS conceptualized the work and participated in the overall study organization, data collection, analysis, manuscript writing. JO participated in the study conceptualization and drafting of the manuscript. BKM was involved in the study organization, data collection, analysis, drafting of the manuscript. All the authors read and approved the final manuscript.
